# Tomatoes, lycopene-containing foods and cancer risk

**DOI:** 10.1038/bjc.2011.59

**Published:** 2011-02-22

**Authors:** G Lippi, G Targher

**Affiliations:** 1 UO di Diagnostica Ematochimica, Dipartimento di Patologia e Medicina di Laboratorio, Azienda Ospedaliero-Universitaria di Parma, Via Gramsci 14, Parma 43126, Italy; 2 Sezione di Endocrinologia, Dipartimento di Scienze Biomediche e Chirurgiche, Università di Verona, Verona, Italy

**Sir,**

We read with interest the recent review article by Key (2011), who concluded that the published results from the epidemiological studies suggest little or no association between the total intake of fruit and vegetables and the risk of common cancers, including colorectal, breast and prostate cancer. Although the association between food intake and cancer is still under intense debate, we believe that there is a growing body of clinical evidence suggesting that certain types of food, for example, those rich in lycopene such as tomatoes, might have beneficial effects on the development of certain cancers, especially prostate cancer.

First and foremost, the most recent expert report issued by the World Cancer Research Fund, together with the American Institute for Cancer Research, has reviewed the strength of the evidence that causally correlates food intake to the risk of several forms of cancer. Basically, it has been concluded that a higher consumption of several plant foods might protect against cancers of various sites. In particular, foods rich in folate may protect against pancreatic cancer, those rich in carotenoids against cancers of the mouth, pharynx, larynx and lung cancer, those rich in *β*-carotene or vitamin C against oesophageal cancer, and those rich in lycopene against prostate cancer (World Cancer Research Fund/American Institute for Cancer Research, 2007).

With regard to the lycopene-containing foods, Giovannucci (1999) carried out a comprehensive review of the epidemiological data linking tomatoes, tomato-based food products and lycopene with the risk of cancer. Interestingly, it was found that the relative risks (RRs) for the comparison between individuals with higher *vs* those with lower dietary intakes of tomato-based food products or between individuals with higher *vs* those with lower plasma levels of lycopene were ⩽0.60, respectively, with a stronger evidence for a potential benefit for prostate cancer. The published data were also suggestive for potential benefits for lung, oesophagus, stomach, colorectal, pancreas, breast and cervix cancers, although a direct cause–effect relationship could not be definitively assessed (Giovannucci, 1999). A large cross-sectional study was conducted in 2002, including patients below age 80 years with incident, histologically confirmed cancer of the oral cavity and pharynx (*n*=754), oesophagus (*n*=304), colorectum (*n*=1953), breast (*n*=2529), ovary (*n*=1031), as well as over 5000 patients below age 80 years with acute, non-neoplastic, non-hormone-related diseases. The analysis of the data showed that the multivariate RR of oral, pharyngeal and oesophageal cancer decreased across subsequent levels of lycopene intake to reach 0.7 (95% confidence interval (CI) 0.4–1.0) for oral and pharyngeal, and 0.7 (95% CI 0.4–1.1) for oesophageal cancer in the highest quintile of intake (La Vecchia, 2002). In the same year, Giovannucci (2002) further reviewed the published epidemiological studies of tomatoes and prostate cancer, and concluded that a high tomato or lycopene consumption might provide up to 40% reduction in the risk of prostate cancer. In a recent meta-analysis of 21 observational studies, Etminan *et al* (2004) confirmed that tomatoes and lycopene-containing foods might have an anti-prostate cancer activity. Compared with non-frequent users of tomato products, the RR of prostate cancer among consumers of high amounts of raw tomato was 0.89 (95% CI 0.80–1.00). For a high intake of cooked tomato products, the corresponding RR was 0.81 (95% CI 0.71–0.92) (Etminan *et al*, 2004). More recently, the putative anti-prostate cancer activity of tomatoes and lycopene-containing foods was further supported by an experimental study, in which tomato diet significantly increased overall survival, delayed progression from prostatic intraepithelial neoplasia to adenocarcinoma, and decreased the incidence of poorly differentiated carcinoma in a model of transgenic adenocarcinoma of the mouse prostate (Pannellini *et al*, 2010). Moreover, in another experimental study, the investigators produced genetically modified tomatoes that overexpressed *Lc* and *C1*, that is, two regulatory genes encoding select transcription factors that control the biosynthesis of anthocyanins in mice. In such study, cancer-susceptible Trp 53−/− mice, which were supplemented with these genetically modified tomatoes rich in anthocyanins, showed a significant extension of their life span (Butelli *et al*, 2008). The outcome of this study is noteworthy, in that it paves the way to further research aimed at establishing whether lycopene, in combination with existing therapies, might be used as a natural compound for prevention and treatment of human cancer.

Although a clear cause–effect relationship between tomato products, lycopene and cancer risk has not yet been established, and further research is urgently needed to draw a definitive conclusion, we believe that the accumulated literature suggests the possibility that a higher consumption of tomato products or lycopene is safe and may have potential benefits for lowering the risk of cancer as well as of other severe pathologies (e.g., atherosclerosis).
